# Supplementary material for: Overview of Nucleocapsid-Targeting Vaccines against COVID-19
Source: Vaccines (Basel). 2023 Dec 3;11(12):1810. doi: 10.3390/vaccines11121810 (PMC10747980; doi:10.3390/vaccines11121810)
Supplement: Supplementary file 1 [file vaccines-11-01810-s001.zip › vaccines-2729032-supplementary.pdf]

vaccines

MDPI

Supplementary Materials

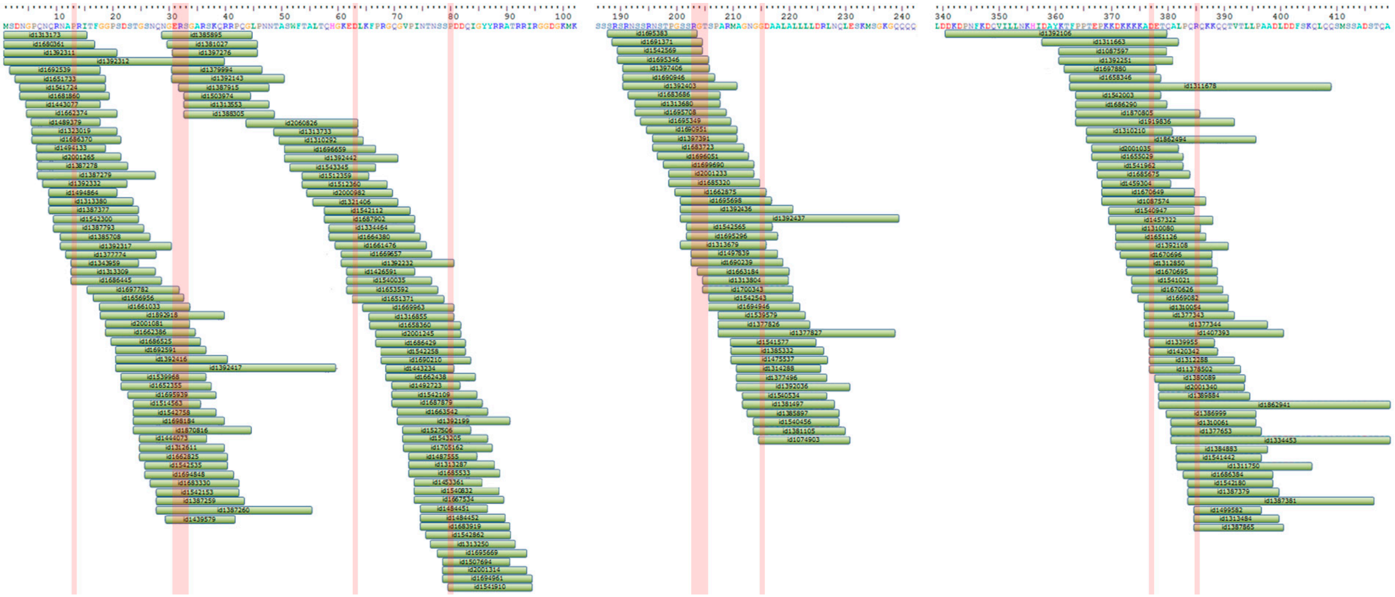

Figure S1. B-cell epitopes of SARS-CoV-2 N protein of B.1 (Wuhan) strain deposited in the Immune Epitope Database which contain variable amino acid residues. The alignment was visualized using the Geneious 10.2.5 Software.
